# Vitrectomy in high myopia: a narrative review

**DOI:** 10.1186/s40942-017-0090-y

**Published:** 2017-10-02

**Authors:** Michele Coppola, Alessandro Rabiolo, Maria Vittoria Cicinelli, Giuseppe Querques, Francesco Bandello

**Affiliations:** 1 Ophthalmology Unit, Azienda Ospedaliera di Desio e Vimercate, Desio, Italy; 2 Department of Ophthalmology, University Vita-Salute, IRCCS Ospedale San Raffaele, Via Olgettina 60, 20132 Milan, Italy

**Keywords:** Degenerative myopia, Epiretinal membrane, Intraoperative optical coherence tomography, Inner limiting membrane, Inverted flap, Macular hole, Pathologic myopia, Posterior staphyloma, Pars plana vitrectomy, Three-dimensional surgery

## Abstract

Pathologic myopia is associated with degenerative changes of the globe, especially at the posterior pole. Eyes affected by pathologic myopia have higher odds to undergo posterior segment surgery and, in those eyes, vitreoretinal surgery is challenging. Many practical tips and tricks can make the surgical procedures simpler, significantly preventing sight-threatening intra- and post-operative complications. Moreover, novel surgical techniques and technological advancements (i.e. ad-hoc instrumentation, minimally invasive vitreoretinal surgery, filters, dye staining, intraoperative optical coherence tomography and 3-dimensional surgery) may play role in highly myopic eyes. The aim of the present work is to review practical tips and tricks, novel surgical techniques and technological advancements.

**Electronic supplementary material:**

The online version of this article (doi:10.1186/s40942-017-0090-y) contains supplementary material, which is available to authorized users.

## Background

Pathologic (or degenerative) myopia is defined as a refractive error >6 diopters (D) and/or axial length >26.5 mm associated with degenerative changes of the eyeball, especially at posterior pole [1]. It has an estimated prevalence of 1–2% in the United States, but it can be even tenfold higher in some districts of Asia and the Middle East [2]. During the past 50–60 years, the prevalence of myopia has been dramatically increasing and some evidence suggest that this trend will worsen with the socio-economic growth of underdevelopment countries [3]. Eyes with high myopia have higher odds of undergoing vitreoretinal surgery due to affections peculiar of myopia (e.g. foveoschisis) or to retinal pathologies (e.g. macular hole [MH], epiretinal membrane [ERM], rhegmatogenous retinal detachment [RD], and vitreous floaters) nonspecific of myopia, but that occur earlier, more frequently or more severely in myopic eyes [1]. Vitreoretinal surgeon’s skills play a primary role in the management of these complications; however, surgery in such eyes is challenging due to higher axial length, posterior staphyloma, thinner and atrophic retina, degenerated vitreous, thinner sclera and abnormal scleral fibers architecture [4].

The aim of this review is to provide practical tips and tricks for vitrectomy in eyes with pathological myopia. Moreover, novel surgical techniques and technological advancements (i.e. ad-hoc instrumentation, minimally invasive vitreoretinal surgery, filters, dye staining, intraoperative optical coherence tomography (iOCT) and 3-dimensional (3D) surgery) will be presented in this narrative review. Table 1 summarizes most of the traditional and novel techiniques to deal with common and challenging issues that the surgeon has to deal with when operating highly myopic eyes.

**Table 1:** Traditional and novel techniques to deal with issues related to high myopia

Issues	Traditional techniques	Novel techniques
Increased axial length	Increased distance between sclerotomies	Ad hoc straight or curved instruments
	Use of 20-G instruments	
	Removal of trocars	
Epiretinal tissues visualization	Chromovitrectomy	iOCT
	Filters	3D
Dye toxicity	Place a substance over the fovea (e.g. PFCL, sodium hyaluronate, autologous blood)	Devices to inject gently the dye (Drip dropper, SideFlo cannula)
	Filters	iOCT
ILM peeling	Start at least 1 DD from the fovea	Diamond Dusted Membrane Scraper
	Start from temporal or inferior quadrants	FINESSE Flex loop
	Elevate preexisting edge using the back of a needle, a MVR blade or vertical scissors	
	PFCL bubble to stabilize retina	
	Lift the flap a bit more than usual	
MH closure	ILM non-peeling	Inverted ILM flap (complete, 270° temporal C-shaped variant, 180° superior variant, Viscoat-assisted)
	ILM peeling	Injection over the hole of autologous platelet-rich plasma, autologous transplantation of ILM membrane, lens capsular flap, neurosensory retina
Shaving vitreous base in eyes with clear lens	Choice of instruments (valved trocars, small G instruments)	Non-contact wide field viewing systems
	Trocar insertion at 4 mm	Ad hoc curved instruments
	Peripheral indentation	Brush the peripheral retina (Diamond Dusted Membrane Scraper, FINESSE Flex loop)
	Hand switching	
	Bending of standard instruments	
Sclerotomy leakage	Biplanar scleral insertion	Triplanar scleral insertion
	Wound construction (Longer tunnel, narrow angle of insertion, parallel to the limbus, bevel-down incision)	27-G instruments
	Sclerotomy massage	Other techniques to close the wound (releasable sutures, tissue glue, polyethylene glycol-based hydrogel bandage, conjunctival cauterization
	Transconjunctival and transcleral absorbable suture	

*G* gauge, *iOCT* intraoperative optical coherence tomography, *3*-*D* three-dimensional, *PFCL* perfluorocarbon liquid, *ILM* inner limiting membrane, *DD* disc diameter, *MH* macular hole

### Axial length

Due to increased axial length and posterior staphyloma, standard instruments may be too short, and therefore unable to reach the retinal surface, especially at the posterior pole [5]. As a consequence, some maneuvers (e.g. fluid or perfluorocarbon liquid removal, ERM and inner limiting membrane [ILM] peeling) may be severely impaired [5]. The surgeon has to verticalize the instruments and apply pressure on the sclera in order to transitorily shorten the axial length of the eyeball [4, 6]. All these elements result in reduced surgeon’s maneuverability, erroneous contact with the viewing system, limited visibility because of corneal folds, longer and more challenging surgical procedures [4, 6]. Some practical tips can be employed to overcome these issues. First, patients with high myopia should undergo axial length measurement and B-scan ultrasonography to identify eventual posterior staphyloma, to adequately plan the intraoperative approach [5]. In order to avoid instruments verticalization and to restore an adequate working angle, the distance between sclerotomies should be increased [6]. Contrary to 25- and 27-gauge (G) devices, 20-G instruments are longer enough to reach the posterior pole, and temporal sclerotomy can be enlarged in order to accommodate such instruments [5]. Alternatively, trocars can be removed during the surgery and the 25-G/27-G microcannula can be directly inserted into the vitreous cavity to gain extra millimeters. Recently, dedicated instrumentation has been developed to deal with longer eyes, such as long-shaft forceps and long cannulae, chosen on the basis of axial length [7]. In order to avoid conflict with the viewing system, curved elongated instruments can be used instead of straight elongated ones [6].

### Epiretinal membrane and inner limiting membrane visualization

ERM and ILM visualization, mobilization, and removal are more challenging in highly myopic than in emmetropic eyes, due to thinner and atrophic retina, more friable tissues, and longer axial length (for the reasons explained above). All these factors may result in incomplete ERM/ILM removal and paracentral iatrogenic macular holes [8, 9]. ILM peeling is a delicate maneuver and some precautions should be adopted to avoid complications. Peeling initiation is the most difficult and dangerous step. Since paracentral iatrogenic macular holes impair visual function only if close to the fovea, ILM peeling should be initiated at least 1-disc diameter away from the fovea [9]. Moreover, ILM dissection should begin at the inferior or temporal quadrants avoiding the superior and nasal quadrants because it can limit the view of the surgical field due to the instrument itself or it can damage papillomacular bundle, respectively. Although many surgeons initiate the dissection using the forceps searching for a natural edge of the ILM, it is advisable to enlarge and elevate a preexisting one or create a new edge in case of absence. On this regard, several instruments commonly found in the in the vitreoretinal surgeon’s armamentarium may be used including the back of a needle, a microvitreoretinal blade or the lower blade of a vertical scissor [10]. These instruments, however, may traumatize outer retinal layers and novel dedicated tools have been proposed to gain an edge, such as the Diamond Dusted Membrane Scraper (Synergetics) [10, 11] and the FINESSE Flex loop (Alcon, Fort Worth, TX, USA) [12]. It has been demonstrated using iOCT the occurrence of micro- and macro-architectural abnormalities during the surgery, which are more pronounced using a direct pinch-and-peel technique, than using the Diamond Dusted Membrane Scraper, which is, in turn, more traumatic than the FINESSE Flex loop [12, 13]. Since myopic ILM and retinal tissues are more fragile, a bubble of perfluorocarbon liquid may be placed on the macular area to stabilize the retina [14]. When performing the peeling, the flap should be lifted a little bit more than usual in order to avoid contact with the wall of the staphyloma.

Several procedures can be adopted to improve visualization and obtain complete removal of ERM and ILM, with minor trauma to the retinal tissue, including chromovitrectomy, filters, iOCT and 3D system.

Chromovitrectomy refers to the use of dyes to stain preretinal tissue and membranes during vitrectomy [15]. Common dyes stain the vitreous (i.e. triamcinolone acetonide, sodium fluorescein and lutein/zeaxanthin), the ERM (i.e. trypan blue, patent blue) and the ILM (indocyanine green [ICG], infracyanine green, brilliant blue, lutein/zeaxanthin + brilliant blue) [15]. Introduced for the first time in 2000, ICG allows a better removal of ILM and better outcomes even in highly myopic eyes, by increasing ILM visualization and stiffness, avoiding its fragmentation and multiple grasping [16]. However, ICG exerts a toxic effect on the retina, especially towards the outer layers and the retinal pigment epithelium, and therefore, nowadays, less harmful vital dyes are preferred [15]. Staining the vitreous is very useful in these patients since many cases of apparent complete posterior detachment have the vitreous cortex attached to the retina, especially in foveoschisis and MH [17]. Due to the vitreoschisis and adherent vitreous cortex on retinal surface, tractional tissues in those eyes are multilayered and heterogeneous consisting of vitreous cortex residuals, ERM and ILM [18]. The surgeon must pay attention to delaminate all the components and chromovitrectomy is extremely useful in this setting. Tissue-specific dyes are of great importance to selectively stain and properly identify the residual vitreous cortex, the ERM and the ILM [19]. Since myopic ILM is firmly adherent, friable and sticky, multiple stainings are advisable to be sure of its complete removal [20].

According to the previous literature, the potential toxic effect of vital dyes arises from the contact between retinal pigment epithelium and the dye itself [21]. Therefore, several substances have been employed in order to create a protective layer over the retina during macular hole surgery, to prevent migration of the dyes into the subretinal space, including sodium hyaluronate [22], perfluorocarbon liquid [23] and autologous blood [24]. In addition, many devices (e.g. drip dropper, or SideFlo cannula) have been proposed to inject the dye gently into the vitreous cavity, avoiding fluidic turbulence with dye migration into the subretinal space [25, 26].

Along with the type of dye, also light filters may improve visualization of stained ILM by improving tissue contrast. Amber filters aid the removal of ILM stained with brilliant blue, whereas a green filter is preferred when ILM is colored with other dyes [27]. These filters have shown to reduce retinal light toxicity [28].

**Fig. 1:**
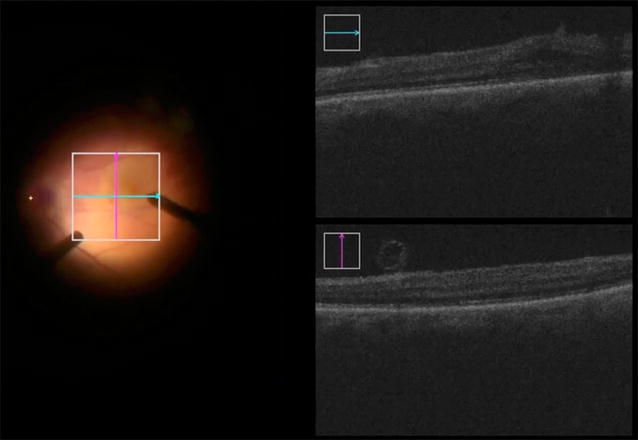
Intraoperative optical coherence tomography assisted inner limiting membrane removal

Microscope-integrated iOCT is an emergent, fascinating technology, which allows the real-time visualization of tissue-instruments interactions. In the setting of macular surgery, iOCT may permit the identification of residual ERM/ILM that would otherwise not have been identified by commercially available microscopes and may guide the surgeon in the intraoperative decision making (Fig. 1) [29].

3D surgery is a newborn technology in which the image is displayed in a large flat monitor letting the surgeon perform different procedures in a heads-up position wearing polarized eyeglasses [30]. Although few papers have been published so far, compared to standard microscope, this technology appears extremely attracting in myopic eyes since it displays images in high definition, and allows good visualization of the vitreous and other tissues using specific channels (e.g. green for vitreous) and modifying image parameters, as digital gain, contrast, and brightness (personal communication, presented at FLOREtina 2017).

Although most of the above-mentioned considerations may be beneficial also in emmetropic eyes, they become extremely precious in pathologic myopia.

The extension of peeling of the ILM membrane in eyes with myopic macular disease is a matter of controversy. On one hand, smaller ILM peeling is more respectful toward retinal nerve fiber layer [31, 32]. On the other hand, ILM is probably the most rigid and non-compliant structure in myopic retina and a wider peeling till to the retinal arcades may allow the relaxation of the elastic retinal tissue [33–36]. Although a complete and wide ILM peeling is beneficial in some kind of myopic macular disease (i.e. MH, foveoschisis-associated MH), it can be harmful in others, including myopic foveoschisis, where an iatrogenic MH may occur due to breakage in the thin central perifoveal tissue [37]. To prevent this complication, it is possible to adopt a center-sparing technique leaving a circular intact ILM fragment around the fovea in order to release the tangential tractions with no mechanical stress on the epifoveolar tissue [38, 39]. Other authors, however, demonstrated that it is possible to get favorable outcomes in myopic foveoschisis even without peeling ILM and, thus, with an easier and safer procedure [40]. A scleral imbrication combined with pars plana vitrectomy without ILM peeling has been described as an additional, effective method to treat myopic foveoschisis [41].

On the contrary, MH and MH-retinal detachment (MHRD) necessitate the ILM peeling in order to release the tractional forces and may have greater benefit from surgical variants, such as the inverted ILM flap technique [35, 42]. In the case of rhegmatogenous retinal detachment, ILM peeling seems to reduce the incidence of epiretinal membrane occurrence [43]; however, more studies are warranted to provide the indication to peel in this specific setting.

### Inverted ILM flap

Many studies have demonstrated that myopic MHs have a worse closure rate than idiopathic ones [44]. This discrepancy has become even more evident with the introduction of the OCT, which has unequivocally shown that elongated eyes have worse anatomic outcomes following MH surgery [45]. Retinal shortening over an elongated sclera, often with posterior staphyloma, has been advocated as a causative factor [46].

In order to compensate for such retinal shortening, an “inverted ILM flap” technique has been attempted, to place the ILM in upside down position inside the MH [46]. In this way, the ILM acts as a scaffold for reactive gliosis and glial cell proliferation and, thus, anatomic MH closure occurs more easily [46]. Alternatively, ILM could act as a barrier preventing the fluid penetration from the vitreous into the macular hole preserving the function of the retinal pigment epithelium [47]. According to the original technique described by Michalewska and colleagues [46, 48], ILM is peeled circumferentially around the hole for about 2 disc diameters leaving the inner boundary attached to the MH edges. Then, the ILM is lifted, rolled and gently massaged inside the MH to cover it with the inverted ILM flap. At last, the fluid-air exchange is performed and the vitreous cavity is filled with air. In the postoperative period, patients are instructed to stay in a prone position up to a week following the surgery. Some variations of the technique have been proposed. If an ERM is present, it could be either peeled off or inverted with ILM [46, 49]. Although some authors use all the ILM flap, it can be shortened using the cutter in order to increase its maneuverability and reduce the chance to have a flap displacement [35, 49]. Alternatively, it is possible to peel the ILM with the exception of the area designated for the flap construction [50]. A mono-layered rather than a multi-layered ILM flap has been proposed with the aim to provide a more regular and physiological scaffold for gliosis [50, 51]. In order to prevent the traumatism to retinal nerve fibers, a modified 270° C-shaped temporal ILM technique has been proposed [52]. Compared to the standard technique, the temporal variant was equally effective, but with a reduction in dissociated optic nerve fiber layer [47]. Additional advantages of temporal variant include the easier procedure, positioning of the flap in proper position with fewer efforts and manipulations, avoidance of ILM flap loss since ILM is not entirely removed. Choi et al. [53] proposed also an 180° superior variant of ILM flap, which does not require the patient to maintain a prone position post-operatively.

After flap position, great attention must be paid to avoid turbulences that could displace the flap from its proper position. Spontaneous flap displacements or detachments are major concerns of the original technique, prolonging the surgery, requiring reposition maneuvers and leading to failure of surgery. On this regard, it has been suggested to perform a fluid-air exchange with low intraocular pressure and passive aspiration [35]. To secure the ILM flap during this step, it is also possible use Perfluoro-n-Octane [50], Viscoat [51] or autologous blood [54]. To further settle the flap, the vitreous cavity can be filled with gas [35, 49, 55].

The anatomic success of inverted ILM flap for myopic MH without retinal detachment ranged from 94 to 100% [35, 46, 55]. A 100% closure rate has been reported even in eyes with axial length ≥30 mm or MH retinal detachment [55, 56]. A comparative analysis disclosed that inverted ILM flap increase of 22 folds the chance to achieve anatomic success compared to complete ILM removal independently from the MH size [35].

The ILM inverted flap is a challenging maneuver and sometimes it cannot be performed due to ILM flap loss during the surgery or in the case of reoperation of eyes with MH that previously underwent complete ILM removal. In such setting, other alternative techniques have been proposed, such as the injection over the MH of autologous platelet-rich plasma [57], the autologous transplantation of ILM membrane [58], lens capsular flap [59] or even a flap of autologous peripheral neurosensory retina [60].

### Retinal detachment

Highly myopic eyes have a tenfold risk increase of experiencing rhegmatogenous RD [61]. Almost 55% of non-traumatic RD occurs in myopic eyes and, in addition, myopia poses at higher risk to develop RD following a blunt trauma [61, 62]. Besides peripheral retina lesions, RD may also arise from posterior paravascular tears and macular holes. Both *ab externo* (i.e. scleral buckle) and *ab interno* (i.e. pars plana vitrectomy) procedures may be applied, but they have a lower success rate in highly myopic eyes [63–65]. In the last decades, the use of scleral buckle declined in favor of pars plana vitrectomy [66].

Due to the increased axial length, thinning and fragility of highly myopic eyes, those eyes are at higher risk of surgical complications including muscle avulsion, vortex vein damage, hemorrhage, subretinal fluid retention, and globe perforation [67, 68]. Since myopic patients are significantly younger than those with idiopathic RD, cataract development is a major concern in those patients [69]. Interestingly, cataract progression risk following vitrectomy is extremely low in patients <50 years old [70]. Scleral buckling carries the lowest risk of cataract onset and, thus, it should be considered as a primary option [71]. In case of pars plan vitrectomy, several precautions should be kept in mind in order to minimize cataract progression in these eyes and to avoid intra-operative cataract formation. Proper choice of instrumentation is the first step. Valved cannulas should be preferred to non-valved ones and infusion volume should be kept low in order to create a controlled, gentle intraocular environment avoiding fluid turbulences. Although controversial, use of small gauge instruments may reduce the incidence of postoperative cataract [71, 72]. Peripheral vitrectomy with meticulous vitreous shaving is highly beneficial in eyes with RD, especially in complicated cases, in order to prevent redetachment or proliferative vitreoretinopathy (PVR) [73–75]. In eyes with cataract, combined phacoemulsification and pars plana vitrectomy is a good option, since it allows a better shaving of vitreous base. In the case of a clear lens, however, several surgical expedients may be applied to avoid lens damage during the peripheral vitrectomy. By improving the visualization of the extreme peripheral retina and ora serrata, non-contact wide field viewing systems have been a technological step forward and are extremely precious in this setting [76]. Peripheral indentation may further aid the visualization of retinal periphery. Trocar cannula should be inserted at 4 mm posterior to the limbus since it allows a higher range of accessibility to the opposite retina without violating the anterior vitreous base [77]. Switching the hand of the vitreous cutter permits to reach the target without crossing the instruments behind the lens base. Curved instruments have been proposed to keep the distance from the posterior lens capsule during the peripheral vitrectomy [77–80]. Alternatively, standard small gauge instruments can be slightly bent to reach the peripheral retina without damaging the lens. Despite all these precautions, total removal of vitreous is arduous in phakic myopic eyes, since intraoperative complete posterior vitreous detachment may be even unachievable in some eyes due to strong vitreoretinal adhesion and posterior shifting of the vitreous base [81]. To further remove vitreous fibers, which may represent a scaffold for proliferation, the Diamond Dusted Membrane Scraper (Synergetics) or the FINESSE Flex loop (Alcon, Fort Worth, TX, USA) may be used to brush peripheral retina. Since myopic eyes have a more posterior vitreous base, a thinner sclera and incomplete posterior vitreous detachment, a scleral buckling in addition to pars plana vitrectomy has been proposed in order to provide a better adherence even after tamponade removal [81, 82]. However, this combined approach may lead to higher incidence of cataract [71] and no strong evidence demonstrated significant advantages over pars plana vitrectomy alone.

Another key point in these eyes concerns the use and the effect of internal tamponades. The irregular contours of the vitreous cavity for the presence of posterior staphyloma and peripheral ectasia may theoretically affect tamponade efficacy. It has been shown that gas tamponades perform better than silicon oil on retrospective studies [83, 84]. Mathematical models illustrated that silicone oil fails to tamponade irregular scleral contours [85]. In addition, silicon oil needs additional surgery for its removal and it may lead to several complications, including emulsion in anterior chamber, cataract, glaucoma, keratopathy, migration into the retina and/or optic nerve, and loss of myelinated optic nerve fibers [86]. For all these reasons, gas tamponades should be preferred to silicone oils in these eyes, except for complicated cases with PVR or for those arising from MHs, which require a long-lasting tamponade [87]. Macular buckling may be performed in case of MHRD refractory to vitrectomy or even as a primary procedure [88–92]. Macular buckle changes the shape of the macula from concave to convex and, in this way, it alleviates antero-posterior and tangential tractions and causes an hyperopic shift [88–92]. Buckle sponges of different material and shape have been proposed [65, 93–95].

Evacuation of subretinal fluid is a further critical step, especially in the setting of MHRD. If a gas was used as a tamponade, subretinal fluid can be left in place since retinal pigment epithelium will pump out the fluid and gas will fill the new space. In case of MHRF, fluid could be drained directly from the hole itself using very small gauge instruments (i.e. 30 G needle); however, this procedure could lead to hole enlargement or damage to its edges. Another option is to perform an inferior retinotomy to evacuate subretinal fluid and this step could be simplified if perfluorocarbon liquid is used, since it may displace peripherally the fluid.

An additional movie file shows a case of inveterate myopic retinal detachment complicated with PVR in a young, highly myopic patient (see Additional file 1).

### Sclerotomy leakage

One of the advantages of minimally invasive vitreoretinal surgery is the possibility to perform transconjunctival sutureless vitrectomy (TSV) thanks to self-healing small-diameter sclerotomies [96]. TSV is associated with shorter operative time, faster postoperative recovery, less postoperative inflammation, and less patient discomfort compared to traditional vitrectomy [96]. However, self-healing sclerotomies not always remain tight, leading to spontaneous fluid loss and early postoperative hypotony; thus, precautionary intraoperative suture placement has been described in 1.3–11.2% of surgeries in general population [97–99]. In a retrospective case series by Woo et al. [99], myopia was an independent risk factor for sclerotomy leakage and early postoperative hypotony. One possible explanation is related to poor wound sealing due to myopic thinner sclera with deranged fibrillar architecture [100]. In addition, vitreous plugging could be less efficient in highly myopic eyes, because of extensive liquefaction [101]. Finally, the pressure on the sclera, exerted by the operators during the procedure in order to reach the posterior pole, could undermine wound sealing.

To avoid sclerotomy leakage, some precautions have been employed. Trocar insertion architecture is of primary importance: the intrascleral tunnel should be built in order to promote apposition of wound margins. Indeed, it has been demonstrated that biplanar oblique-perpendicular sclerotomies have lower incidence of leakage, conjunctival bleb formation, and postoperative hypotony, compared to straight cuts [102, 103]. Moreover, longer tunnels are structurally more secure than shorter ones [104]. Due to trigonometric laws, the narrow is the angle between the instruments and sclera, the longer is the incision wound; therefore, it is preferable to puncture the sclera with a narrow-angle [104]. Beyond angle of incision, also trocar blade bevel position has an impact on tunnel length, as bevel-down incision leads to longer tunnel than bevel-up cuts [105]. Finally, incision should be parallel to the limbus in order to avoid cutting of scleral fibers, which could impair the healing of the ocular surface [106, 107]. Some authors [108, 109] suggested a tri-planar sclerotomy similar to cataract tunnel with a precut using a sharp instrument (e.g. stiletto blade) prior to trocar insertion with the theoretical advantages of having a longer tunnel, sharp margins, and increased valve effect.

Even with those precautions, sclerotomy leakage may eventually occur, due to myopic sclera altered architecture [99]. Although some of them can be managed with prolonged sclerotomy massage [108, 110], additional maneuvers are usually performed to prevent post-operative hypotony. A single transconjunctival and transscleral absorbable suture is a popular choice [111]. Although this manoeuver is relatively simple, cost-effective and requires no peritomy, it has some drawbacks (i.e. foreign body sensation and astigmatism) [112, 113]. In order to avoid such complications, other techniques have been proposed including releasable sutures [114, 115], use of tissue glue [116], polyethylene glycol–based hydrogel bandage [117, 118], and conjunctival cauterization [111, 119]. Although routine placement of sclerotomy sutures has been advocated in eye at high risk of leakage (e.g. highly myopic ones) [120], few pieces of evidences may be found in the current literature. In our practice, we usually place sutures in case of leakage after a careful examination of the sclerotomy and sclerotomy massage; in these eyes, however, the threshold to suture should be low.

Less than 10 years ago, Oshima et al. [121] introduced 27-G vitrectomy with an incision size of 0.40 mm. Since it does not require biplanar beveled trocar insertion and carries a very low risk of hypotony, 27-G vitrectomy offers extreme advantages in eyes with high myopia [122]. However, its employment may be limited in eyes with extremely high axial length and/or posterior staphyloma (see axial length paragraph) and a preoperative planning of should take in account all the above-mentioned variables.

### Methods of literature search

A Pubmed engine search was carried out using the terms “myopia” paired with “vitrectomy” and “vitreoretinal surgery”; “posterior staphyloma”; “intraoperative optical coherence tomography”; “three-dimensional vitreoretinal surgery”; “chromovitrectomy”; “transconjunctival sutureless vitrectomy”; “internal limiting membrane”; “retinal detachment”. All studies published in English up to July 2017 irrespective of their publication status were reviewed and relevant publications were included in this review.

## Conclusions

To conclude, despite modern surgical devices, myopic eyes still represent a challenge for vitreoretinal surgeons. Many practical tips and tricks can make the surgical procedures simpler, significantly preventing sight-threatening intra- and post-operative complications. Future perspectives in this field include the shortening of the learning curve for each procedure, the introduction of novel technologies and new instruments in order to reach satisfying postoperative results.

## Additional file

**Additional file 1.** 27-G inveterate myopic retinal detachment. The additional movie file shows a pars plana vitrectomy in a young, phakic patient for inveterate retinal detachment complicated by proliferative vitreoretinopathy using 27 gauge instruments.

## Abbreviations

D: diopters
ERM: epiretinal membrane
G: gauge
ICG: indocyanine green
ILM: inner limiting membrane
iOCT: intraoperative optical coherence tomography
MH: macular hole
MHRD: macular hole-retinal detachment
PVR: proliferative vitreoretinopathy
RD: retinal detachment
TSV: transconjunctival sutureless vitrectomy
3-D: three dimensional
